# Dual-Domain Reporter Approach for Multiplex Identification of Major SARS-CoV-2 Variants of Concern in a Microarray-Based Assay

**DOI:** 10.3390/bios13020269

**Published:** 2023-02-13

**Authors:** Francesco Damin, Silvia Galbiati, Nicola Clementi, Roberto Ferrarese, Nicasio Mancini, Laura Sola, Marcella Chiari

**Affiliations:** 1 National Research Council of Italy, Institute of Chemical Sciences and Technologies “G. Natta”, 20131 Milan, Italy; 2 Complications of Diabetes Units, Diabetes Research Institute, IRCCS San Raffaele Scientific Institute, 20132 Milan, Italy; 3 Laboratory of Medical Microbiology and Virology, Vita-Salute San Raffaele University, 20132 Milan, Italy; 4 Laboratory of Medical Microbiology and Virology, IRCCS San Raffaele Hospital, 20132 Milan, Italy

**Keywords:** SARS-CoV-2, microarray, biosensor, molecular diagnostics, microarray-based assay, COVID-19, genotyping, variant of concern, VOCs

## Abstract

Since the emergence of the COVID-19 pandemic in December 2019, the SARS-CoV-2 virus continues to evolve into many variants emerging around the world. To enable regular surveillance and timely adjustments in public health interventions, it is of the utmost importance to accurately monitor and track the distribution of variants as rapidly as possible. Genome sequencing is the gold standard for monitoring the evolution of the virus, but it is not cost-effective, rapid and easily accessible. We have developed a microarray-based assay that can distinguish known viral variants present in clinical samples by simultaneously detecting mutations in the Spike protein gene. In this method, the viral nucleic acid, extracted from nasopharyngeal swabs, after RT-PCR, hybridizes in solution with specific dual-domain oligonucleotide reporters. The domains complementary to the Spike protein gene sequence encompassing the mutation form hybrids in solution that are directed by the second domain (“barcode” domain) at specific locations on coated silicon chips. The method utilizes characteristic fluorescence signatures to unequivocally differentiate, in a single assay, different known SARS-CoV-2 variants. In the nasopharyngeal swabs of patients, this multiplex system was able to genotype the variants which have caused waves of infections worldwide, reported by the WHO as being of concern (VOCs), namely Alpha, Beta, Gamma, Delta and Omicron variants.

## 1. Introduction

SARS-CoV-2 is the virus responsible for the COVID-19 pandemic, which emerged in 2019 and has since spread worldwide, infecting more than 600 million people and causing more than six million deaths [1]. SARS-CoV-2 has a positive-stranded RNA genome of ~30 kb size, encoding 29 proteins; it belongs to the Coronaviridae family [2]. Despite the presence of a proofreading activity, SARS-CoV-2 evolves over time by fixing mutations to increase its transmission in humans and to circumvent host adaptive immunity. The evolution rate of SARS-CoV-2 is lower compared to those of other RNA viruses due to the presence of an acquired enzyme which is capable of excising the erroneous mutagenic nucleotides incorporated by their RNA polymerase, maintaining relative fidelity during replication and transcription [3,4,5,6]. Indeed, SARS-CoV-2 has a mutation rate of about two non-synonymous mutations per month. Due to its relatively slow mutation rate, in the early days of the rapid development of a vaccine, the immune escape of emerging variants was not considered a priority, considering the huge number of deaths during the first pandemic waves [7,8]. Amongst the variables that influence the selection of immune-escape variants, long viral shedding, observed especially in immunocompromised people, contributed to the emergence of escape mutations, examples of which are the Gamma, Beta, and Omicron variants [9,10]. The World Health Organization and the Centers for Disease Control and Prevention (CDC) have identified SARS-CoV-2 lineages, designated as “variants of concern” (VOCs) [11], which have mutations along the entire genome. However, clinically significant mutations have been identified mainly in the Spike protein (S protein), a trimeric envelope glycoprotein mediating both target cell recognition and fusion processes. Any alterations in the structure of the S protein could modulate different processes involved in viral infection and eventually determine the selective advantage for the virus, thus affecting the kinetics of infection of different variants [12]. VOCs have increased transmissibility compared to the clade responsible for the Wuhan epidemic, and some of them have been related to an increase in the severity of the disease such as, for example, the Delta variant. The first vaccine formulations were based on the prototype Spike sequence of the Wuhan strain (Wuhan-Hu-1) [13,14,15,16,17]. However, due to the adaptive selection of VOCs carriers of new mutations on the spike protein, new vaccines containing drifted spike variants are now accessible [18]. The five variants causing the majority of infections globally are B.1.1.7 (the UK or Alpha variant), B.1.351 (the South African or Beta variant), B.1.1.28 (P.1, the Brazilian or Gamma variant), B.1.617.2 (the Indian or Delta variant) and a larger group of the lineage B.1.1.529 (BA.1-BA.2-BA.5 or Omicron1, Omicron2, and Omicron5 variants). All these sequences contain several single point mutations in the S protein, more precisely in the receptor-binding domain (RBD), including the E484K, E484Q, E484A, and L452R, mutations [19,20]. In B.1.1.7, B.1.351, BA.1, and BA.5, single point mutations and small deletions were also identified in the N-terminal domains (NTD) of the Spike protein. Therefore, in order to allow a timely adaptation of public health interventions, accurate monitoring of the appearance and distribution of these VOCs in a timely manner is extremely important. At present, two methods are principally applied to identify SARS-CoV-2 variants: the sequencing of the full viral genomes by Sanger sequencing and next-generation sequencing (NGS) [21,22,23,24], and quantitative reverse transcription PCR (RT-qPCR) [25]. Although genome sequencing is extremely accurate and can recognize any mutations in a sample, routine genomic testing is costly, time-consuming, and requires specialized tools and interpretation. In contrast, PCR-based techniques, such as quantitative PCR (qPCR) [25], melting-temperature RT-PCR [26], and CRISPR-Cas13a-based transcription amplification [27], identify single or few mutations simultaneously (4 or 5 maximum, due to limited number of available fluorescence channels) [28]. There is, therefore, an urgent need to develop a simple, sensitive, and faster detection method to accurately identify multiple SARS-CoV-2 circulating VOCs simultaneously.

DNA microarrays, based on the use of different specific probes, offer an ideal tool for identifying variations in PCR products.

In a previous work [29], our group proposed the CovidArray, a method for detecting SARS-CoV-2 in nasopharyngeal swabs featuring high sensitivity, accuracy, and multiplexing capability based on microarray technology. The approach consists of a solid-phase hybridization of fluorescently labeled amplicons on polymer-coated silicon chips, after RNA extraction, and reverse transcription with the aim of detecting SARS-CoV-2 N1 and N2 markers in nasopharyngeal swabs. In this study, we introduce a microarray-based assay that can distinguish the known viral variants present in clinical samples by simultaneously detecting mutations in the Spike protein gene. To reach the level of specificity necessary to discriminate between sequences that differ by only a single nucleotide, we applied an optimized version of the method previously used by our group in the field of liquid biopsy [30] for the genotyping of mutations in genes (*KRAS*, *BRAF*, and *NRAS*) involved in colorectal cancer. In this new approach, the viral nucleic acid, extracted from nasopharyngeal swabs after RT-PCR, hybridizes in solution with specific Dual-domain oligonucleotide reporters. The domains complementary to the S protein gene sequence comprising the mutation form hybrids in solution which are directed by the second domain (“barcode” domain) at specific locations on coated silicon chips. The amplicons captured on the array surface are detected by means of their Cy3-labeled reverse primers. Using nasopharyngeal swabs, this multiplex system, with high specificity, can genotype the variants which have caused waves of infections worldwide, reported by the WHO as being of concern (VOCs), namely the Alpha, Beta, Gamma, Delta and Omicron variants. During the development of this assay, the continuous evolution of SARS-CoV-2 has led to the emergence of new Omicron subvariants (BA.4/5, BQ.1, XBB, BF.7, BF.11, etc.) which are no longer distinguishable only by the characterization of the mutations in the S gene included in our assay. However, it is noteworthy that the flexibility and the modularity of our system allows the detection of all the new variants simply by adding new capture probes to the surface of the array and the corresponding Dual-domain reporters into the hybridization mixture. In this case, a Dual-domain reporter sequence will be designed in order to also encompass new mutations.

## 2. Materials and Methods

### 2.1. Materials and Reagents

Copoly(DMA-NAS-MAPS) (MCP-4) was purchased from Lucidant Polymers Inc., Sunnyvale, CA, USA. Ammonium sulfate ((NH_4_)_2_SO_4_), ethanolamine and 20× standard saline sodium citrate (SSC) solution (3 M sodium chloride, 0.3 M sodium citrate, pH 7.0), and sodium dodecyl sulfate (SDS) were obtained from Sigma Aldrich (St. Louis, MO, USA). The oligonucleotides were synthesized by Metabion International AG (Steinkirchen, Germany). Untreated silicon/silicon oxide chips with 100 nm thermal grown oxide (15 × 15 mm) were supplied by SVM, Silicon Valley Microelectronics Inc. (Santa Clara, CA, USA). Chips were pretreated using a HARRICK Plasma Cleaner, PDC-002 (Ithaca, NY, USA), connected to an oxygen line. Spotting was performed using a SciFLEXARRAYER S12 (Scienion, Berlin, Germany). Fluorescence images were obtained using InnoScan 710 (Innopsys, Carbonne, France). Data intensities were extracted with the Mapix software (Version 8.2.7), and data analysis was performed for each experiment.

### 2.2. Clinical Samples

Nasopharyngeal swabs were performed using FLOQSwabs^®^ (COPAN Cat#306C) in UTM^®^ Universal Transport Medium (COPAN Cat#306C). All samples were analyzed by the Laboratory of Medical Microbiology and Virology at IRCCS San Raffaele Hospital and stored at −80 °C until processing. Additionally, 200 μL of each swab sample was lysed and used for RNA extraction.

### 2.3. RNA Extraction

RNA was extracted from nasopharyngeal swabs using the ELITe InGenius^®^ system (ELITechGroup, Puteaux, France) following the manufacturer’s instructions.

### 2.4. Reverse Transcription and PCR Conditions

We performed a triplex PCR in which we simultaneously amplified two different regions of the Spike protein gene to correctly identify the VOCs and the human β-actin gene as a control of RNA extraction, retrotrascription, and amplification. The primer sequences are shown in Table 1.

The triplex PCR was performed in 20 µL of reactions containing 5 µL of RNA, 250 nM and 1500 nM of primers for Amplicon1 and 2 respectively, 20 nM of primers for human beta-actin gene, and 40X One-Step RT mix and 5X One-Step PCR Mix with UNG (Solis BioDyne Tartu, Estonia). The cycling conditions were as follows: 50 °C for 15 min, 95 °C for 10 min; 35 cycles at 95 °C for 5 s, 60 °C for 10 s, 72 °C for 10 s, and finally, 72 °C for 1 min.

### 2.5. Silicon Chip Coating and Microarray Preparation

The silicon chips (15 × 15 mm) were activated by a treatment with oxygen plasma (15 min) and then coated with MCP-4 in accordance with the protocol provided by the manufacturer.

As capture probes, we selected twelve different oligonucleotides (reported in Table 2), taken from those published in [31]. The array includes capture probes utilized to detect the following: the S-gene deletion (S:delH69V70), present in the Alpha (UK, B.1.1.7), Omicron1 (BA.1), and Omicron5 (BA.5) VOCs; the RBD mutations S:T20N and P26S, present in Gamma (Brazil, P.1); S:D80A, present in Beta (South Africa, B.1.351); S:L452R, present in the Delta (India, B.1.617.2), and Omicron5 (BA.5) VOCs; S:E484A, present in Omicron1 (BA.1), Omicron2 (BA.2), and Omicron5 (BA.5); and finally, S:E484K, present in the Beta and Gamma variants. In addition to these sequences, capture probes corresponding to the “wild-types” derived from the original “Wuhan-Hu-1” strain were included. As an internal control of RNA extraction, retrotranscription, and amplification, we added to the array an oligonucleotide probe specific for the human β-Actin gene. The sequences of the spotted probes are reported in Table 2.

The capture probes were immobilized as reported in [29]. To increase the material bonded to the chip, each printed spot consists of 10 drops. The reproducibility of the microarray fabrication and the quality control of the DNA microarrays were evaluated as described in [32].

After the spotting step, the chips were incubated overnight, and then all remaining reactive groups of the coating polymer were blocked as previously described [31].

### 2.6. SARS-CoV-2 Variants Specific Hybridization in Solution

A mixture of seven Dual-domain reporters specific to the mutations for the SARS-CoV-2 Alfa, Beta, Gamma, Delta, and Omicrons VOCs, and five Dual-domain reporters, corresponding to the original “Wuhan-Hu-1” sequences, was prepared by diluting in water, in equimolar amounts, all the reporters from a 100 µM stock solution to obtain a final concentration of 1 µM each. The sequences of the Dual-domain reporters are reported in Table 2.

For the mutations T20N and P26S, due to the proximity of the mutations, a single Dual-domain reporter, including both mutated nucleotides, was used.

The hybridization in solution was carried out in a final volume of 25 µL containing 20 µL of the fluorescently labeled PCR, 2.5 µL of 20X SSC hybridization buffer (final concentration 2×), and 2.5 µL of the Dual-domain mixture (final concentration: 0.1 µM of each reporter). The hybridization mixture was placed in a thermocycler where it was first heated at 95 °C for 5 min. Then, it underwent a stepwise gradient of temperature ranging from 55 °C to 48 °C. In particular, the mixture was kept for 2 min at 55, 54, 53, 52, 51, 50, 49, and 48 °C.

### 2.7. Microarray Hybridization and Image Scanning

After the thermal gradient, the mixture was quickly centrifuged at 13,000 rpm for 10 s and spread onto the spotted silicon chips. The microarray hybridization, the washing steps, and the data analysis was performed as reported in [29].

## 3. Results and Discussion

### 3.1. Design of the Assay for SARS-CoV-2 Variants

**Figure 1:**
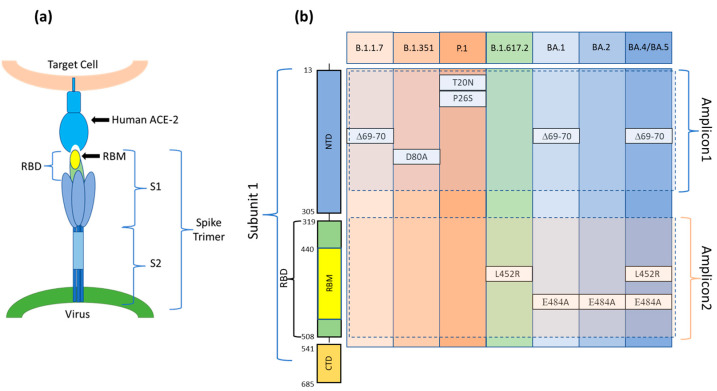
(**a**) The Spike protein is made up of three spike monomers which form a homotrimeric structure (Spike trimer) on the SARS-CoV-2 surface. Each spike monomer is divided into two subunits, i.e., S1 and S2, connected at the Furin cleavage site. During viral fusion, the receptor binding domain (RBD) in the S1 subunit binds to the host cell target receptor ACE2, whereas the S2 domain enables membrane fusion. (**b**) A schematic diagram and aminoacidic sequence of the S1 subunit of the SARS-CoV-2 S protein. Positions of the mutations present in each of the variants and included in Amplicon1 (positions 23 to 289 nt in the S protein coding sequence) and Amplicon2 (positions 1215 to 1545 nt in the S protein coding sequence). S1: Ectodomain S1 subunit; S2: Ectodomain S2 subunit; NTD: N-terminal domain; RBD: Receptor binding domain; RBM: Receptor binding motif; CTD: C-terminal domain.

Viruses such as SARS-CoV-2, due to genetic mutations or viral recombination which occur during genome replication, evolve continuously, resulting in different variants of the same virus. SARS-CoV-2 variants are classified into variants of interest (VOI), variants of concern (VOC), and variants under surveillance (VUM) [33]. Among these, variants of concerns (VOC)s are those for which there is clear evidence indicating a significant impact on transmissibility, severity, and/or immunity that could affect the epidemiological situation. As of 30 August 2022, five VOCs (Alpha, Beta, Gamma, Delta, and Omicron) have been designated by the WHO. Each variant is associated with multiple mutations in the Spike protein gene, primarily in the Receptor Binding Domain (RBD), enhancing the viral binding affinity with the host cell’s ACE2 receptor (Figure 1a) [34]. Many mutations are shared among VOCs, but some mutations, or a combination of these, are characteristic of a particular VOC and can be used to specifically determine the type of variant. The gold standard method to diagnose COVID-19 is real-time quantitative reverse transcription-polymerase chain reaction (qRT-PCR). However, many other molecular methodologies, such as RNA sequencing (RNA-seq), droplet digital reverse transcription-polymerase chain reaction (ddRT-PCR), reverse transcription loop-mediated isothermal amplification (RT-LAMP), and clustered regularly interspaced short palindromic repeats (CRISPR), have been implemented [35]. RNA-seq is required for finding SARS-CoV-2 variants; however, it is not useful for rapid screening and requires specialized equipment and expertise. ddRT-PCR is a sensitive methodology, but it also requires expensive equipment and consumables. LAMP is a simple and fast technique that works in isothermal conditions and does not require costly lab equipment. However, the primer design and the optimization of the process are not simple and rapid to achieve. In addition, the technique identifies the presence/absence of the virus without specifically recognizing variants. To our knowledge, to date, only one group has developed an RT-LAMP assay for specific discrimination of the SARS-CoV-2 Delta variant detecting the R203M mutation [36]. Lastly, CRISPR is a novel, rapid, low-cost, and highly sensitive assay. However, its major disadvantage is the off-target effect whose consequences are poor signaling and potential for misinterpretation of the results [37]. Furthermore, a deep knowledge of the viral genome is required to devise tests for emerging variants using CRISPR, which hinders the timely development of tests [37]. The decisive disadvantage of these technologies is that they target single or few mutations per reaction (limited to the number of available fluorescence channels). In our microarray-based assay, the variants of SARS-CoV-2 are identified by probing the RT-PCR products derived from the viral RNA (extracted from clinical samples which have already been characterized) for the presence or absence, in a specific combination, of six single point mutations at the same time (Figure 1b).

**Figure 2:**
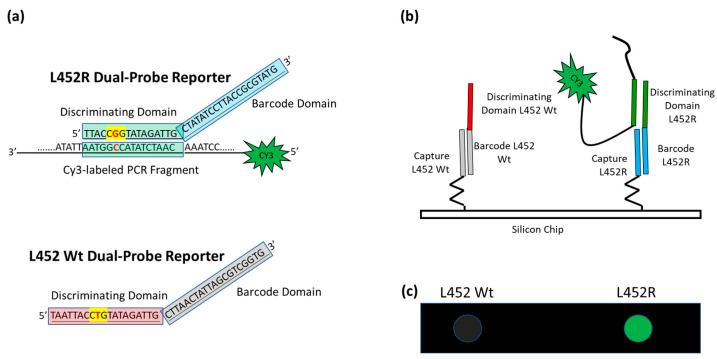
Schematic of the assay. (**a**) In the case of L452R mutation (the nucleotide triplet encompassing the point mutation is highlighted in yellow with the mutation in red), the Discriminating Domain of the L452R Dual-domain reporter hybridizes in solution with the Cy3 labeled single strand PCR, whereas the L452 Wt Dual-domain reporter does not. (**b**) The different Barcode Domains in the 3′ portion of the Dual-domain reporters (the different colors represent difference in the sequences) direct the labeled PCR to different positions on the array by surface hybridization with the corresponding capture probes. (**c**) The incorporation of Cy3-Reverse primers into the PCR amplicons allows the exact mutation present in the protein S gene to be detected by fluorescence scanning. The image shows the highlighting of the spot corresponding to mutation L452R, whereas the spot corresponding to L452 Wt remains dark.

A schematic of the assay is presented in Figure 2. In our approach, the viral RNA is extracted from the nasopharyngeal swabs and then retrotranscribed and amplified with a one-step reaction.

To avoid the formation of secondary structures that could hide the sequences that enclose the mutations, we amplified not the entire S gene but only the two sequences that comprise all the mutations under examination: Amplicon1 (positions 23 to 289 nt in the S protein coding sequence) and Amplicon2 (positions 1215 to 1545 nt in the S protein coding sequence) (Figure 1b).

The amplicons of the sequences encompassing the mutation sites, fluorescently labeled with Cy3 linked to the 5′ of the reverse primers, are hybridized in solution with a mixture of specific oligonucleotide reporters, called Dual-domain reporters (Figure 2a), whose sequence consists of two domains. The 5′ domain (discriminating domain) is complementary to the SARS-CoV-2 genome sequence comprising the mutation, whereas the 3′ domain is a “barcode” sequence (barcode domain) that recognizes complementary oligonucleotide probes (capture probes) spotted at specific locations on the silicon chip. After the incubation in solution, the Cy3 labeled PCRs are captured on the surface of the array through the hybridization of the 3′ barcode domain of the Dual-domain reporters with the corresponding capture probes on the silicon chip surface, thereby revealing the type of variant through a specific positional signature (Figure 2b).

The key factor in achieving accurate viral variant identification is separating mutation detection from surface capture. Indeed, the efficacy of surface DNA hybridization compared with solution DNA hybridization suffers from some limitations, such as the electrostatic repulsion between DNA strands on the surface, and the steric hindrance between tethered DNA probes [38], which may impair the specific hybridization of sequences that differ by a single base. Furthermore, to achieve correct genotyping in standard microarray assays where the specific capture reporters are arrayed onto surface, the hybridization on the surface must be carried out at a specific temperature. Although careful optimization of the length and sequence of the probes can partially overcome this problem, it is difficult to find optimal conditions for all mutations. In our approach, the hybridization of the PCRs with the Dual-domain reporters is carried out in solution in a thermal gradient ranging from 55 °C to 48 °C. In this gradient, every dual-domain reporter binds to an amplicon under optimal conditions. When the Cy3-labeled PCR coupled to the Dual-domain reporters is captured onto the microarray surface, less stringent conditions are required, and the same temperature is used for all barcodes. Finally, the surface chemistry that allows for covalent bonding of the capture probes to the silicon chip surface with high binding capacity and low non-specific adsorption [39], as well as the use of a fluorescence-enhancing silicon/silicon oxide substrate [40], are fundamental features of the system. The density of the immobilized capture probe was measured in a previous work using an interferometric technique named Interferometric Reflectance Imaging Sensor (IRIS) [32,41]. In particular, when spotting a 10 μM solution of the oligonucleotide, the mean density of the immobilized probe ranges between 1–3 ng/mm^2^.

### 3.2. Specificity of the Variant-Microarray Technology

Many of the variants that have appeared since the end of 2020 share defining aminoacid mutations. The five VOCs of the lineages B.1.1.7 (Alpha variant), B.1.351 (Beta variant), P.1 (Gamma variant), B.1.617.2 (Delta variant), and B.1.1.529 (Omicron variant containing the sub-lineages BA.1, BA.2 and, BA.5) include 8, 9, 10, 10, and 32 mutations in the S protein, respectively [42]. Some of these mutations are of particular importance for immune evasion and for the binding affinity of the viral S protein to the human ACE2 receptor [43,44,45].

For our assay, we chose defined mutations whose presence, alone or in combination with each other, uniquely defines the VOCs under study. For this purpose, the delH69V70, T20N, P26S-D80A, L452R, and E484A mutations, shown in Figure 1b, were chosen. In addition to these mutations, we designed a Dual domain reporter for the E484K mutation. However, this mutation is shared between the Beta and Gamma variants.

The first step in the development of the assay was to verify the functionality and the specificity of the Dual-domain reporters in discriminating the mutations present in the products of the RT-PCR of the virus RNA derived from nasopharyngeal swabs.

**Figure 3:**
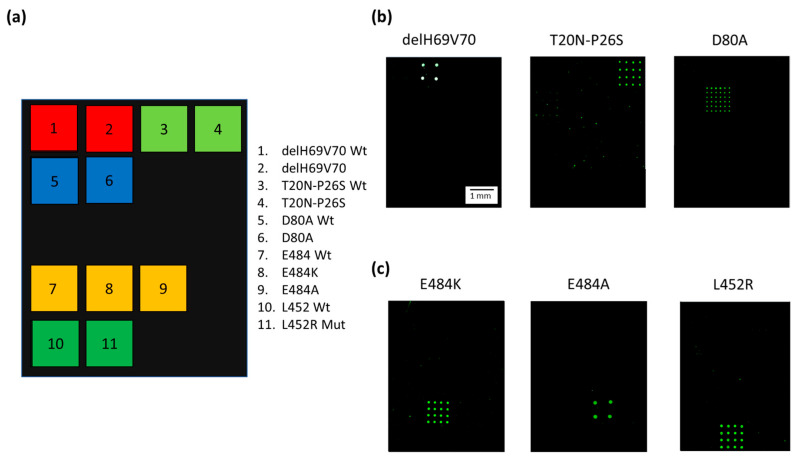
(**a**) Spotting schematic of the array. Silicon chips coated with MCP-4 are used as substrates for the covalent binding of amino-modified capture probe oligonucleotides arrayed at discrete locations. Each position in the grid identifies an individual capture probe address corresponding to the mutations described in the legend. (**b**) Cy3 fluorescence images of three different silicon chips. Each array is hybridized with an individual Cy3 labeled PCR (Amplicon1, positions 23 to 289 nt in the S protein coding sequence) incubated with Dual-domain reporters corresponding to delH69V70 and T20N-P26S and D80A mutations respectively. (**c**) Cy3 fluorescence images of three different silicon chips. Each array is hybridized with an individual Cy3 labeled PCR (Amplicon2, positions 1215 to 1545 nt in the S protein coding sequence) incubated with dual probe reporters corresponding to E484K, E848A, and L452R mutations. The spots are 150 µm large with a pitch of 350 µm. The scale bar (1 mm) is the same in (**b**,**c**).

Figure 3a shows the spotting scheme of the microarray. The capture oligonucleotides complementary to the 3′ Barcode sequence of the Dual-domain reporters specific for the variant mutations were spotted together with the capture probes for the “wild-type” (wt) derived from the original “Wuhan-Hu-1” strain with no mutations. Figure 3b presents Cy3 fluorescence images of three different silicon chips, each hybridized with Amplicon1 products incubated with the single Dual-domain reporter specific for the mutation present in the sample with a known genotype. The Dual-domain reporters specific to the delH69V70, T20N-P26S, and D80A mutations present in the Alpha, Beta and Gamma variants recognize specifically and without cross-hybridization the mutated sequences included in Amplicon1, while the 3′ barcode domains of the reporters direct the Cy3 labeled PCR to the specific capture probes onto the chips. Figure 3c shows the Cy3 fluorescence images of the amplification products, i.e., Amplicon2, including the L452R, E484A, and E484K mutations (present in the Delta, Omicron and Beta/Gamma variants respectively), incubated with the corresponding Dual-domain reporters and then spread over three different chips. Again, the signals are mutation specific, with no cross-hybridization.

Different subarray patterns, with 4 spots on some chips and 16 spots on others, were adopted during the assay development. However, the different spot patterns do not affect the correct assignment of the genotype of the samples. The white color of the spots in the image (Figure 3b, delH69V70) indicates a high level of hybridization and, therefore, saturation of the fluorescence signal. The presence of saturated signals prevented us from quantitating the target analyte which, in any case, was not within the scope of this work.

### 3.3. Discrimination of the Different Sub-Variants of the Omicron Lineage

**Figure 4:**
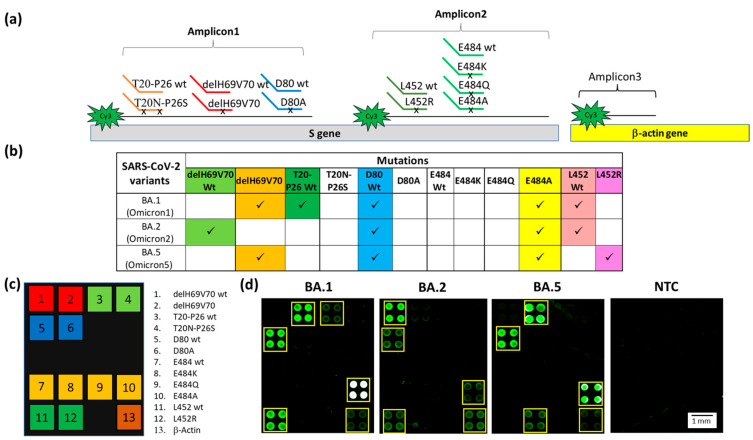
(**a**) Schematic representation of the Dual-domain reporters used in the assay and the Cy3 labeled strands generated in the PCR multiplex assay. A small black x on the discriminating domain of the Dual-domain reporters indicates the presence of the mutations. The human β-actin amplicon (Amplicon3) hybridizes directly to the capture probe bound to the microarray surface. (**b**) Specific mutations signature for the Omicron BA.1, BA.2 and BA.5 sub-variants. The mutations detected in the assay are shown in the columns and sub-variants are shown in the rows. Each of the Omicron sub-variants can be identified by a specific pattern of mutations combination. (**c**) The spotting schema of the array. (**d**) Cy3 fluorescence image of four silicon chips. Each chip is hybridized with a triplex Cy3-labeled PCR (Amplicon1, 2 and 3) from samples carried the BA.1, BA.2, BA.5 sub-variants and a No Template Control (NTC) with no DNA, incubated with the whole set of Dual-domain reporters. The yellow squares in the images were used to highlight more easily the spots corresponding to the presence of hybridization. The spots are 400 µm large with a pitch of 800 µm.

Before the emergence of the B.1.1.529 lineage (Omicron variant) with its most widespread BA.1, 2, and 5 sub-variants, a single PCR (Amplicon1 or Amplicon2) was able to discriminate among the Alpha, Beta, Gamma, and Delta variants. After the emergence of the Omicron variants, two amplicons were required to discriminate the different sub-variants. To simplify but, at the same time, make the assay workflow more robust, a multiplex PCR reaction was developed. The discrimination of the different sub-variants of the Omicron lineage is based on the simultaneous presence, or absence, of some mutations already included in previous panel, i.e., the delH69V70 deletion (Amplicon1) and the L452R and E484A mutations (Amplicon2) [46,47], as detailed in Figure 4b. Hence, there is a need to detect mutations in different sites within the S protein gene with a single assay. To this end, we performed a multiplex PCR reaction simultaneously amplifying the sequences corresponding to Amplicon1, 2, and the Human β-actin gene (Amplicon3). The multiplex PCR was hybridized in solution with the mixture of all specific Dual-domain reporters (7 mutant reporters and 5 wt reporters) in a stepwise gradient of temperature ranging, after a denaturation step (5 min at 95 °C), from 55 °C to 48 °C. The temperature gradient is based on the theoretical melting temperature of the Dual-domain reporters, adjusted by trial and error in order to obtain a sufficiently strong signal.

Particular attention was given to codon 484, a mutational hot spot site of various mutations, namely E484A (present in all Omicron subvariants), E484K (present in Beta and Gamma variants), and E484Q (present in lineage B.1.617.1 or the Kappa variant, which is not considered a VOC). In order to have a complete genotyping of this site, we designed the capture probes and the Dual-domain reporters for this last mutation (see Table 1). The results of the multiplex assay for the identification of the Omicron sub-variants are shown in Figure 4. The PCRs and the Dual-domain reporters used in the assay are indicated in Figure 4a. In Figure 4b, the mutations indicative of the presence of a given variant are reported. Two mutations, delH69V70 deletion and E484A, characterize the BA.1 sub-variant. The absence of the mutations associated with the other variants included in the assay is revealed by the fluorescence signal of the subarray corresponding to the original “Wuhan-Hu-1” strain for the T20N-P26S (T20-P26 wt), D80A (D80 wt) and L452R (L452 wt) mutations (Figure 4d). In the sample carrying the BA.1 subvariant, the four spots corresponding to the E484A mutation are white due to saturation of the fluorescence signal caused by a high level of hybridization. The BA.2 sub-variant is correctly genotyped by the presence of the fluorescence signal corresponding to the E484A mutation and by the signals corresponding to the wild-type of all the other mutations (Figure 4d). Interestingly, there was a lack of signal corresponding to the T20-P26 wt, caused by the presence in the BA.2, and 5 sub-variants of the T19I mutation (not included in the panel of the array) near the recognition site of the Dual Domain reporter for T20-P26 wt, which prevents the same reporter from hybridizing with the Amplicon1. The BA.5 sub-variant is characterized by the presence of signals in correspondence of the delH69V70 deletion and to the E484A and L452R mutations (Figure 4d). As expected, the NTC does not show significant fluorescence. The positive control (human β-actin gene) is detected in all samples. The latter amplicon does not require discrimination between similar sequences; therefore, it is captured directly by a surface capture probe. Using the appropriate Dual probes, it was also possible to identify in a multiplex assay samples of B.1.351 (Beta variant), P.1 (Gamma variant), B.1.617.2 (Delta variant). The presence of non-uniform spots in Figure 4d is due to the non-uniform drying of the spots themselves and is exacerbated by the low concentration of the target analyte. Despite the use of an optimized printing buffer that mitigates the coffee-ring effect, the phenomenon is not fully suppressed. However, it does not affect the identification of the mutation [48].

### 3.4. Clinical Verification

To validate the variant-microarray assay with clinically relevant samples, we blind analyzed the RNA extracted from fourteen nasopharyngeal swabs previously subjected to solid-phase extraction and RT-qPCR at IRCCS San Raffaele (Milan, Italy). We performed a triplex amplification reaction to obtain the Amplicon1, 2, and 3 for each of the 14 samples. The amplification products were subjected to hybridization in solution, in temperature gradient, and with the Dual-domain reporter mixture, and subsequently hybridized to the silicon chips, using one chip for sample.

**Figure 5:**
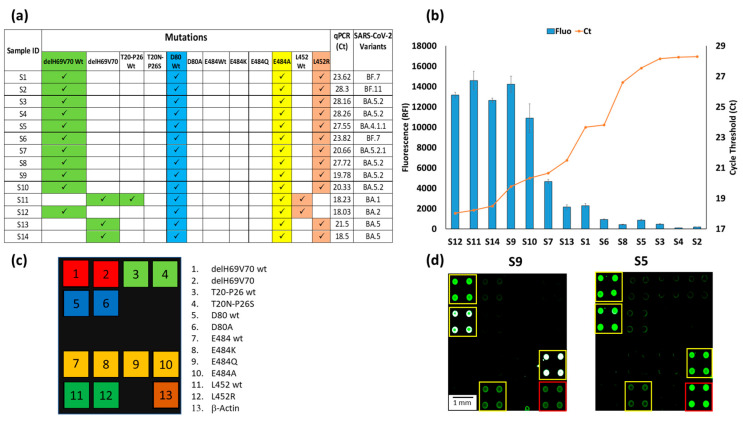
(**a**) Mutations signature of the sample S1-14, the corresponding Ct of the qPCR of the same samples and the identification of the SARS-CoV-2 subvariants based on genome sequencing. (**b**) Plots of the correspondence between the relative fluorescence intensity (blue bars) of the hybridization of the 14 chips with samples S1-14, with the number of Cycle Threshold (Ct) of the corresponding qPCR (red line) of the same 14 samples. The blue bars are the average of the relative fluorescence intensity (RFI) of the 4 spots (2 × 2 subarrays) of the 484A capture probe subarray. The error bars are the standard deviations of the fluorescence intensity of the subarray. (**c**) The spotting schema of the array. (**d**) Example of Cy3 fluorescence image of two silicon chips. Each chip is hybridized with a triplex Cy3-labeled PCR (Amplicon1, 2 and 3) from samples S9 and S5. The yellow squares in the images were used to highlight the spots corresponding to the presence of hybridization. The red square indicates the hybridization of the Amplicon3 (β-actin). The spots are 400 µm large with a pitch of 800 µm.

Hybridizations produced specific signatures on the arrays (Figure 5a) that allowed the identification of the SARS-CoV-2 variants. In this case, the signatures of samples S11, 12, 13, and 14 were characteristic of the Omicron subvariants, with S11 corresponding to the BA.1, S12 to the BA.2, and S13 and S14 to BA.5 subvariant. These results are in agreement with those obtained by RT-qPCR. In contrast, for the samples from 1 to 10, the absence of the delH69V70 deletion and the simultaneous presence of the E484A and L452R mutations led us to suspect the presence of other types of Omicrons subvariants, i.e., different from the BA.1, 2 or 5. Our finding was confirmed by the sequencing method, conducted in San Raffaele hospital, which identified various subvariants (listed in Figure 5a) derived from the Omicron variant. These subvariants, as verified by means of online resources (Overview of SARS-CoV-2 Variants (https://www.nih.gov/)), are characterized by the mutations identified in our assay and, moreover, differ from each other and from the Omicron subvariants already analyzed in this work by mutations in different viral genes outside the S gene or by other mutations in the S gene not yet included in our array.

The analyses carried out in the laboratories of San Raffaele hospital on the viral RNA extracted from nasopharyngeal swabs highlighted the presence of samples within a wide range of viral loads with consequent different qPCR threshold cycles (Ct), i.e., from 18.03 Ct in S12 up to 28.3 in S2. The graph in Figure 5b shows the inverse proportionality between the fluorescence obtained with our assay and the qPCR threshold cycles. The bars represent the average fluorescence intensity of the spots corresponding to the E484A mutation, i.e., the characteristic mutation of the Omicron subvariants are always present in all chips. In this case, the relative fluorescence intensity (RFI) reaches a maximum with samples with low Ct or high viral load (S12, S11, S14, S9) and gradually decreases with increasing Ct, until it becomes significantly lower, albeit remaining perfectly detectable with a Ct of around 30 (S4, S2). These results confirm that the microarray-based assay not only determines the type of virus variant but also provides a qualitative indication of its viral load. Figure 5d shows the fluorescence images of two samples (S9 and S5) with high (Ct 19.78) and low (Ct 27.55) viral loads. In a previous work, the sensitivity of the microarray system for detecting the presence of the virus (but not for discriminating its variants) in a nasopharyngeal swab was assessed using serial dilution of synthetic N1 and N2 genes [29]. The sensitivity was high (about 1 copies/µL), i.e., comparable to that declared by various qPCR kit manufacturers. However, the target of this work is not the sensitive detection of the virus, but the simultaneous detection of several point mutations. The fluorescence intensity of the spots corresponding to the various mutations provides a qualitative indication of the viral load in the samples, as demonstrated in the Figure 5b, where a correspondence between qPCR Ct and fluorescence signal is demonstrated. The white color of the spots in the image corresponding to sample number 9 (S9) indicates a high level of hybridization and, therefore, saturation of the fluorescence signal.

## 4. Conclusions

This article reports on a microarray-based assay which is capable of detecting and genotyping known SARS-CoV-2 variants of concern (VOCs) in clinical samples, such as nasopharyngeal swabs, via the simultaneous detection of mutations in the Spike protein gene. The Dual-domain probe approach allowed the multiplex detection of point mutations in solution thanks to the temperature gradient, and the use of Dual-probe sequences with a domain complementary to capture probes spotted on the surface of the silicon chips. The method utilizes characteristic fluorescence signatures to unequivocally differentiate, in a single assay, different known SARS-CoV-2 variants and accurately classifies patient samples identified in parallel by Whole Genome Sequencing (WGS). The total time required for multiplex variant detection is approximately 4.5 h including sample preparation, Reverse Transcription-PCR (steps common with other standard methods), solution and microarray hybridization, and result analysis. The multiplexing capability is the key factor that allows the reduction of analysis time (one test for all variants) and cost due to the reduction of reagents and hands-on time.

It must be said that the current platform, with its instrumentation requirements (PCR thermal cycler, fluorescence scanner), is not suitable for use in environments outside well-equipped hospital analysis laboratories, e.g., pharmacies or at home. A possible obstacle to using the current platform in analytical laboratories is the high degree of manual work required to perform the test. However, there are no conceptual obstacles to integrating the assay into an automated platform, and we are now working to enable the entire workflow, from viral RNA extraction to detection, to be performed in a microplate equipped with a fluorescence reader for microplates. With the emergence of several variants of concern, and their potential importance in the context of vaccine use, this approach is inexpensive, rapid, and scalable, and provides a useful tool for epidemiological surveillance.

Finally, an important feature of our approach is its versatility, which makes it possible to follow the evolution of SARS-CoV-2 variants by adding new Dual-domain probes to systems and the corresponding capture probes spotted on the array. Furthermore, the method can be applied in other clinical settings such as, for example, in microbiological, oncological, and pharmacogenomic contexts, wherever the discrimination of single nucleotide is necessary.

## Figures and Tables

**Table 1:** Primers sequences for SARS-CoV-2 and human β-actin gene amplification.

	Primer Sequences	Fragment Lenght (bp)
Amplicon1 (Spike gene)	Forward: 5′-TGCCACTAGTCTCTAGTCAGTGT-3′ Reverse: * 5′-CTCAGTGGAAGCAAAATAAACACCATC-3′	266
Amplicon2 (Spike gene)	Forward: 5′-GATGAAGTCAGACAAATCGCTCCA-3′ Reverse: * 5′-AGAAAGTACTACTACTCTGTATGGTTGGTAA -3′	330
Amplicon3 (human β-actin gene)	Forward: 5′-GCGAGAAGATGACCCAGATCATG-3′ Reverse: * 5′-AGAGGCGTACAGGGATAGCA-3′	89

* The Reverse primers are labeled with Cyanine 3 (Cy3) in 5′ end.

**Table 2:** Sequences of spotted probes and reporters.

SARS-CoV-2 Variant Mutations	Capture Probes ^1^ (5′→3′)	Dual Domain Reporter Sequences
S: delH69V70	ggctcacgtcttatttgggc	* CCATGCTATA TCTGGGACCAATGGTACTAAG-^†^ gcccaaataagacgtgagcc
Wild-Type	cgagcacttaacattagagc	* CATGCTATACATGTCTCTGGGACCAATGGT-^†^ gctctaatgttaagtgctcg
S: T20N-P26S	gcctcgggcaaacgactaa	* AACAGAACTCAATTACCCTCT-^†^ tttagtcgtttgcccgaggc
Wild-Type	taatctaattctggtcgcgg	* ACCAGAACTCAATTACCCCCT-^†^ ccgcgaccagaattagatta
S: D80A	attgaccaaactgcggtgcg	* TGCTAACCCTGTC-^†^ cgcaccgcagtttggtcaat
Wild-Type	tcttctagttgtcgagcagg	* AGGTTTGATAACCCT-^†^ cctgctcgacaactagaaga
S: L452R	catacgcggtaaggatata	* TTACCGGTATAGATTG-^†^ ctatatccttaccgcgtatg
Wild-Type	caccgacgctaatagttaag	* TAATTACCTGTATAGATTG-^†^ cttaactattagcgtcggtg
S: E484A	atcgtacttggcactggagt	* ATGGTGTTGCAGGTTT-^†^ actccagtgccaagtacgat
S: E484K	aatgctcgggaaggctactc	* ATGGTGTTAAAGGTTT-^†^ gagtagccttcccgagcatt
S: E484Q	tcttgacggaaaggtagac	* TGGTGTTCAAGGT-^†^ tgtctacctttccgtcaaga
Wild-Type	atcccgtgagtcgatggttt	* TGGTGTTGAAGGT-^†^ aaaccatcgactcacgggat
β-actin sequence	tgagaccttcaacaccccagccatgta	

^1^ The spotted capture probes are amino modified in 5′-end; * sequences which hybridize to the SARS-CoV-2 PCR (the variant base is in bold and red; a wide central space indicates where the deletion is for the delH69V70); ^†^ The tails of the reporter oligonucleotides which hybridize to spotted capture probes. The denomination “Wild-Type” is for the original version of the virus: “Wuhan-Hu-1” strain.

## Data Availability

Data is contained within the article.
